# Assessing Health Threatening Problems among Nursing or Midwifery Students during the Clinical Education Course in Turkey

**Published:** 2019-01

**Authors:** Besey ÖREN, Neriman ZENGIN

**Affiliations:** 1. Department of Midwifery, Faculty of Health Sciences, Health Science University, Istanbul, Turkey; 2. Department of Midwifery, Faculty of Health Sciences, Istanbul University-Cerrahpaşa, Istanbul, Turkey

**Keywords:** Occupational exposure, Injuries, Nursing, Health occupations

## Abstract

**Background:**

This study determined the occupational exposure and health problems experienced by nursing and midwifery students during their clinical internships.

**Methods:**

The study population consisted of 1719 nursing and midwifery students studying at the health sciences faculties of six Turkish universities in 2016. Data were collected using a questionnaire prepared by researchers, namely Occupational Exposure and Health Problems in Clinical Environment Questionnaire, and the State-Trait Anxiety Scale. The data were analyzed using Mann Whitney U test, Kruskal Wallis variance analysis, and Spearman correlation analysis. Significance was accepted in a 95% confidence interval and a level of *P*<0.05.

**Results:**

The students had a mean age of 20.86 yr (1.72), and 48.6% had midwifery and 51.4% had nursing major. More than 17.8% of the students were experienced occupational exposure during their clinical internships. Total score for students was 2.15 ±0.71. The scores of the students examined for exposure to hazards and verbal violence was 2.13 ± 1.17, for needle stick injury it was 2.10 ± 1.13. In addition, when the scores of the students’ health problems were examined, insomnia 3.57 ± 1.22, low back pain 2.84 ± 1.29, shoulder or arm pain 2.68 ± 1.29 were determined statistically. There was a positive significant relationship between the mean clinical occupational hazardous exposure and health problems and state-trait anxiety scores (*P*<0.01).

**Conclusion:**

Approximately one-fifth of the students were exposed to occupational hazardous at the hospital while they were on their clinical internship programs. Students rarely experience occupational exposure, but often suffer from insomnia, sometimes musculoskeletal pains (back, shoulder arm, neck), rarely have skin problems.

## Introduction

The nursing and midwifery students are the largest groups in the healthcare students. Therefore, the clinical education of these students is very important, as they are crucial in providing healthcare. Nursing and midwifery education in Turkey is 4600 h (1, 2), these programs consist of 50% of clinical training (1–3). Students can participate the patient care in clinical education on the supervision of the instructor. The number of schools offering nursing and midwifery undergraduate education in Turkey has increased in recent years. Unfortunately, the number of instructors did not increase at the same level. This has created the risk of students’ exposure to occupational hazards due to shortage of instructors (4).

Clinical practice is an intensive aspect of contemporary nursing and midwifery curriculum (5–7) and is a required core component of their education (7, 8). This education takes place in the various clinical settings, which build the foundation for the ongoing development of students’ critical thinking and decision-making skills; as well as developing professional practice competency (9, 10). However, the clinical settings consist of highly occupational hazardous and risky environments with human resources from different qualities and quantities, complicated work processes, intense use of technology (11, 12). Because of these hazardous environments, the health of many trainees and workers are compromised each year (12, 13). Health workers may be exposed to hazardous environments and drugs such as chemotherapy drugs and gases, (14–16) violence (12, 17), needlestick injuries (NSI)(18–20), skin problems, latex allergies (21), musculo-skeletal system problems (back injuries) (22, 23), cancer from working at night (24), loss of hearing (25) and mental health problems (26). Especially midwifery and nursing students can experience stress (27, 28) and anxiety (29) because of their underdeveloped skills, lack of information, assignments with deadline, being exposed to occupational hazardous as other professional health workers in the clinical environment (30). Moreover, students seek to learn new protocols or due to nature of job, they perform practices outside of the common procedure (31).

The researchers conducted the studies about contaminated or non-contaminated needlestick injury (18, 19, 32–34) around the world, the subjects of sleeplessness, musculoskeletal system disorders, hospital infections, and varicosity, which are all occupational health problems and often encountered in health workers. However, the number of studies investigating all these problems is limited in students.

This cross-sectional study was conducted to assess occupational exposure and health problems among nursing or midwifery students during the clinical education course, and to examine the relationship between health problems and anxiety.

## Methods

### Participants

Health and Sciences Faculties of 57 Turkish universities have midwifery and nursing departments. A survey was conducted of students at 14 universities among the 57 universities, which have these departments. The universities and students were randomly but homogenously selected at different regions and classes. The study was conducted in the first half of 2016 and a survey mailed to the selected faculties. After brief introduction by the faculty members to the students, the surveys were filled out and mailed backed. The number of students participated in the survey was 4800. The number reflected entire population and just 1719 students completed the survey as without any error and without missing parts. The rest of the surveys were excluded.

### Instruments

Data were collected using a questionnaire prepared by researchers, namely Occupational Exposure and Health Problems in Clinical Environment Questionnaire (35), and the State-Trait Anxiety Scale (STAI)(36).

The questionnaires were designed to ask about the socio-demographic characteristics of the students, during their university study years, the services where they performed clinical applications, their status regarding being exposed to occupational hazardous exposure in the clinical environment, and whether they felt safe during clinical internships.

### The Occupational Hazardous Exposure and Health Problems in Clinical Environment Questionnaire

The form was developed by Sarıçam in 2012 in Turkey. This form consists of two sections which assess occupational hazardous exposure, health problems (Table 1) and protection from them. Twelve items evaluating occupational hazardous exposure and health problems were used. Five of the items in the form related to the occupational hazardous exposure and seven items to the experienced health problems in the clinical environment. (35). This 12-question form encompassed the situations of being injured by needlestick, being exposed to the effects of chemotherapy drugs, being exposed to verbal or physical violence or harassment from the relatives of patients, neck, back, shoulder and arm pain or sleeplessness, varicosity, experiencing skin related health problems because of latex gloves, and viral infections stemming from the hospital. The terms were scored in a 5-way Likert type manner with the answers always=5, often=4, sometimes=3, rarely=2, and never=1. The score originally varies between 12 and 60, and a higher score indicates higher exposure to risks stemming from the clinical practice environment. Cronbach’s alpha confident of the scale was 0.59. The Cronbach alpha coefficient of the form in the current study was found to be 0.80.

**Table 1:** Occupational hazardous exposure and health problems (N=1719)

***Occupational hazardous exposure***	***Mean (Sd)***
• Were you ever exposed to verbal violence by patient relatives in the hospital?	2.13 (1.17)
• Did you experience needlestick injury?	2.10 (1.13)
• Were you ever exposed to the negative effects of chemotherapy drugs?	1.32 (0.87)
• Were you ever harassed by patient relatives?	1.31 (0.85)
• Were you ever exposed to physical violence by patient relatives in the hospital?	1.28 (0.82)
• Did you experience a viral infection stemming from the hospital?	1.67 (1.11)
* Health problems *	
• Did you experience sleeplessness?	3.57 (1.22)
• Did you experience back pain that negatively affected your health?	2.84 (1.29)
• Did you experience shoulder or arm pain that negatively affected your health?	2.68 (1.29)
• Did you experience neck pain that negatively affected your health?	2.52 (1.30)
• Did you experience skin problems because of latex gloves?	2.45 (1.46)
Total	2.15 (0.71)

sd=standard deviation, (5) always, (4) often, (3) sometimes, (2) rarely, and (1) never.

High score indicates increased hazards and health problem

### State-Trait Anxiety Scale (STAI)

The STAI scale, was implemented for the Turkish population in 1995 and measures the anxiety level of individuals 14 and older. The STAI consists of two subscales, i.e. (1) the state and (2) the trait anxiety. State anxiety (A-State) is defined as the fear an individual feel because of a stressful situation. Trait anxiety (A-Trait) is the predilection that an individual experiences anxiety. The total score can theoretically vary between 20 and 80. A high score means a high level of anxiety while a lower score shows a lower level of anxiety (36). A Cronbach’s alpha coefficient reported for the A-State Scale of 0.92-0.81 and 0.85-0.71 for the A-Trait Scale (37, 38). In this study, Cronbach alpha for A-State was 0.90 and for A-Trait was 0.82.

### Procedure

The study was conducted in six health sciences faculties from three regions. After the students were informed on the aim of the study by department chairs or responsible lecturers, the students that agreed to participate were given questionnaires. The forms were retrieved via postage and fees were paid by the researcher.

### Data analysis

Statistical evaluation was performed using the IBM SPSS 21 software (Chicago, IL, USA). Descriptive statistics such as frequency, mean, standard deviation, and percentage were used to analyze all the variables under study. In the comparisons between binary groups, the Mann Whitney U test was used, and in the comparison of groups with three or more variables, the Kruskal Wallis variance analysis was used. The relationship between health problems and anxiety levels was examined using Spearman correlation analysis. Significance was accepted in a 95% confidence interval and a level of *P*<0.05.

### Ethical considerations

Before the study, permission was taken from the local board of ethics (Medipol University Ethical Board: Date: 30-03-2015 and No:108400987-157). Students were informed before participation, and participation was on a voluntary basis.

## Results

The mean age of the students was 20.86 (1.72). There was almost a 50/50 ratio between students that study to become a midwife or nurse (48.6%; n=841 studied midwifery and 51.4%; n=878 studied nursing). Ten percent of the students were freshmen, 35.2% were sophomores, 30% were juniors, and 24.8% were seniors. Most of the students had clinical internships in internal diseases (64.8%), surgery (65.4%), and obstetrics (56.0%) clinics. Midwifery students had their clinical internships in the obstetrics clinics (76.0%) and outpatient services (59.9%) while most of the nursing students had their internships in internal diseases (89%) and surgery (77.0%) clinics. 17.8% of the students were exposed to occupational hazard in the clinical environment (Table 2).

**Table 2:** The characteristics of the students

***Variable***		***Midwifery Mean(SD)/median***	***Nursing Mean (SD)/median***	***TOTAL Mean (SD)/median***
Age(yr)		20.89 (1.66) / 21	20.83 (1.77) / 21	20.86 (1.72) / 21
Clinical internship (hours)		14.59 (7.76) / 16	16.07 (6.67) / 16	15.36 (7.25) / 16
		n (%)	n (%)	n (%)
		841 (48.9)	878 (51.1)	1719
Gender	Female	826	711 (81.4)	1537 (90.4)
	Male	0	163 (18.6)	163 (9.6)
Grade	1 ^ st ^	102 (12.3)	68 (7.8)	170 (10)
	2 ^ nd ^	278 (33.7)	320 (36.6)	598 (35.2)
	3 ^ rd ^	274 (33.2)	236 (27.0)	510 (30.0)
	4 ^ th ^	172 (20.8)	250 (28.6)	422 (24.8)
Clinical internship fields	Internal diseases	333 (39.6)	781 (89.0)	1114 (64.8)
	Surgery	449 (53.4)	676 (77.0)	1125 (65.4)
	Intensive care unit	165 (19.6)	306 (34.9)	471 (27.4)
	Pediatrics	271 (32.2)	188 (21.5)	459 (26.7)
	Obstetrics	639 (76.0)	323 (36.8)	962 (56.0)
	Out-patient	504 (59.9)	221 (25.2)	725 (42.2)
Occupational hazardous exposure	Yes	137 (8.05)	167 (9.82)	304 (17.88)
	No	704 (83.7)	711 (81.0)	1416 (82.4)
Training on occupational exposures	Yes	499 (59.3)	512 (58.3)	1011(58.8)
	No	342 (40.7)	366 (41.7)	708 (41.2)

Mean clinical occupational exposure and health problem score of the students are 2.15±0.71. When the student’s occupational hazardous exposure examined, they rarely experienced verbal violence by patient’s relatives (2.13±1.17), and NSI (2.10±1, 13).

However, when the student’s health problems were monitored, the scores were as follow: mostly sleeplessness (3.57 ± 1.22), rare pain in the back (2.84 ± 1.29), in the shoulders and arms (2.68 ± 1.29), pain in the neck (2.52 ±1.30) and skin problems related to the use of gloves (2.45 ± 1.46) (Table 1).

A significant difference was found between the years of study in school and the occupational hazardous exposure and health problems stemming from the clinical internships. The mean score of the seniors (2.26±0.67) was significantly (*P*<0.001) higher than the mean scores of the freshmen, sophomores, and juniors (1.96±0.76, 2.08±0.72, 2.21±0.69 respectively). Furthermore, a positive significant relationship was found between the mean clinical occupational hazardous exposure and health problems scores and the A-Trait (r=.124 *P*<0.001), State-A (r=0.103, *P*<0.001) and anxiety scores (Table 3).

**Table 3:** The statues of the students regarding experiencing occupational exposures and health problems in the clinical internship according to certain characteristics (N=1719)

***Characteristics***		***Occupational hazardous exposures and health problems Mean (Sd)***		
Grade	1 ^ st ^	1.964 (0.76)	Kw- χ ^ 2 ^ =45.754	* P * =0.000
Year	2 ^ nd ^	2.078 (0.72)		
	3 ^ rd ^	2.214 (0.69)		
	4 ^ th ^	2.261(0.67)		
Department	Midwifery	2.169 (0.72)	z= −.860	* P * =0.390
	Nursing	2.136 (0.69)		
Gender	Female	2.157(0.69)	z=−1.760	* P * =0.078
	Male	2.115 (0.83)		
Training	Yes	2.132 (0.71)	z=−1.616	* P * =0.106
	No	2.181 (0.70)		
Occupational hazardous exposure of the clinical internship and health problems			A-State	A-Trait
Were you ever exposed to verbal violence by patient relatives in the hospital			r=0.112 p<0.001	-
Did you experience back pain that negatively affected your health?			r=0.141 p<0.001	-
Did you experience shoulder or arm pain that negatively affected your health?			r=0.108	-
			* P * <0.001	
Total score			r=0.103	r=0.124
			* P * <0.000	* P * <0.000

Sd= Standard deviation, Kw- χ ^2^ = Kruskal Wallis Test, z= Mann -Whitney U

## Discussion

This study presents health-threatening problems among nursing or midwifery students during the clinical education course. 17.8% of the students were exposed to at least one occupational hazardous exposure. Students are rarely exposed to verbal violence and needlestick in the clinical period and reported health problems associated with the clinic were mostly insomnia, rarely musculoskeletal pain and skin problems.

In Turkey, 74.4% of health workers were found to be exposed to violence for reasons stemming from the health care system, with most of the violence being verbal (39). In another study in Turkey, similarly, verbal violence (91.1%) was reported to be more prevalent than physical violence (33%) (40). Students encounter violence in the clinical area and have insufficient information on the management of the problem (41). In our study, rarely of the students stated that they were exposed to violence (Table 1). The reason behind this low rate may be students not dealing with patients actively as much as professional health workers and the students lacking information on the definition of violence.

Exposure to bloodborne pathogens due to NSI resulting in serious occupational diseases constitutes a potential risk factor for nursing and midwifery students (42–44). Students are especially susceptible to NSI’s because of lack of workplace safety awareness and limited clinical experience. The frequency of NSI in studies conducted in Turkey varies between 19.4% and 52.5% (18, 45–48), while in other countries the frequency of NSI occurs between 13.9% and 59.9% (19, 49–51). In our study rarely needlestick cases were seen (Table 1). The lower rate in Turkey compared to other studies may be tied to the fact that students in Turkey do not participate in active application in many fields because of the rising number of students, they receive a mandatory course regarding workplace safety trainings, as well as increased number of simulation laboratories, increased lab hours in nursing and midwifery curricula and decreased clinical internship hours. Training given to students decreases NSI (34). In our study, 67.7% of the students exposed to clinical risk received training (Table 2).

In our study, the most frequent health problem of the students is sleeplessness (3.57±1.2) (Table 1). Nursing students reported that sleep problems were common, headache, severe depression and poor quality of life were effective in the formation of sleep problems, smoking, physical pain, and prevalence is between 26.7% and 56.7% (52). In our study, students often experience insomnia. This may not be a health problem directly related to clinical practice. However, this finding may not be evaluated in the study but may be related to the beginning of the clinical practice in the early morning hours, the physical exhaustion of students throughout the day, the stressful clinical setting, individual characteristics of the students, and the high level of homework related to the clinic. Our results are consistent with the literature in terms of the frequency of insomnia problem.

The second health problem of the students was musculoskeletal problems. Musculoskeletal problems were reported to start in nursing students before even starting the occupation (30). This result supports the findings and shows that the health of students gets disrupted by the working environment just like professional health members.

In our study, the third health problem was “experiencing skin problems because of latex glove use”. In studies, worldwide latex sensitivity is reported to be 1% in the general population and 5%–12% for occupational latex sensitivity (53). In a study with health care students, the frequency of skin problems related to latex glove use was reported to be 4%, with the rate being lower in students compared to health workers (54). Findings of the study are similar to the literature.

In this study, senior students experienced more health problems. Most of the senior students spent an average of 32 h/wk in the clinic due to internship applications which could count for the higher rate of health problems. The stress levels of sophomore nursing students were found to be greater than freshmen nursing students because of increased time of clinical work during sophomore year (55).

Nurses and midwife’s students emphasize the importance of clinical experience and environment as an effective factor in students’ anxiety (37, 38, 56, 57). In our study, the clinical anxiety levels of the students were found to be mild or medium (State anxiety 42,03±6.85; Trait anxiety 47,51±6.62), with their health problem status and their state and trait anxiety being related on a positive low level (Table 3).

Health workers may have mental health problems such as anxiety and depression related to the work environment (58, 59). Occupational exposure has also been reported to have psychological effects (31, 60) In nurses experiencing exposure to occupational hazards, the prevalence of anxiety is higher (26), and nurses experience feelings of anxiety, anger, and guilt (60, 61). Similarly, there are similar mental problems in the studies conducted in the students (31, 62). In our study, anxiety levels increased as a result of experiencing severe exposure, waist, arm and shoulder pain in the clinical environment and the fear of experiencing such stressful situations they live (31, 62). However, unlike the literature (31), there is no relationship between NSI and anxiety (Table 3).

In addition, occupational exposure and health problems total score and state-trait anxiety increased in our study. As well as professional nurses (26, 61) there was a positive correlation between exposure to hazards and health problems and anxiety among students. These results are consistent with the literature.

A limitation of this study was primarily based on students’ memories or recall experiences. Moreover, the results of this study are limited to the studied sample and cannot be generalized.

## Conclusion

The increase in clinical internships led to more health problems. Waist, arm and shoulder pains, as well as violence situations, increase correlates with student’s anxiety level increase. As the frequency of occupational exposure and health problems increases, the state-trait anxiety levels also increase. Further studies are needed to define the risks encountered by nursing and midwifery students in the clinical field and the consequences on their health. Because a few reports have shown the factors affecting the health of nursing and midwifery students in the clinical application process. Further research will support nursing and midwifery training in the prevention of risks and exposures

## Ethical considerations

Ethical issues (Including plagiarism, informed consent, misconduct, data fabrication and/or falsification, double publication and/or submission, redundancy, etc.) have been completely observed by the authors.
